# Quality of life and cochlear implant: results in adults with postlingual hearing loss^☆^

**DOI:** 10.1016/j.bjorl.2017.06.005

**Published:** 2017-07-05

**Authors:** Aline Faria de Sousa, Maria Inês Vieira Couto, Ana Claudia Martinho-Carvalho

**Affiliations:** Universidade de São Paulo (USP), Faculdade de Medicina, São Paulo, SP, Brazil

**Keywords:** Cochlear implantation, Quality of life, Hearing loss, Adult, Deafness, Implante coclear, Qualidade de vida, Perda auditiva, Adulto, Surdez

## Abstract

**Introduction:**

Considering the variability of results found in the clinical population using a cochlear implant, researchers in the area have been interested in the inclusion of quality of life measures to subjectively assess the benefits of the implantation.

**Objective:**

To assess the quality of life of adult users of cochlear implant.

**Methods:**

A cross-sectional and clinical study in a group of 26 adults of both genders, with mean duration of cochlear implant use of 6.6 years. The Nijmegen Cochlear Implantation Questionnaire and the generic World Health Organization Quality of Life questionnaire were sent electronically.

**Results:**

The best assessed domain in the quality of life assessment for the cochlear implantation questionnaire was the social domain, whereas for the quality of life questionnaire it was the psychological domain. The variables, gender, time of cochlear implant use and auditory modality did not influence the results of both questionnaires. Only the variable level of education was correlated with the environment domain of the quality of life questionnaire. The variable telephone speech comprehension was associated with a better perception of quality of life for all the domains of the specific questionnaire and for the self-assessment of quality of life in general.

**Conclusion:**

From the users’ perspective, both questionnaires showed that cochlear implant brought benefits to different aspects related to quality of life.

## Resumo

**Introdução:**

Diante da variabilidade de resultados clínicos encontrada na população usuária de implante coclear, pesquisadores da área têm se interessado pela inclusão de medidas de qualidade de vida para avaliar de maneira subjetiva os benefícios do implante coclear.

**Objetivo:**

Avaliar a qualidade de vida de adultos usuários de implante coclear.

**Método:**

Estudo transversal e clínico em um grupo de 26 adultos, de ambos os gêneros, com tempo de uso médio do implante coclear de 6,6 anos. Foram utilizados o questionário específico *Nijmegen de Implantes Cocleares* e o questionário genérico *World Health Organization Quality of Life*, enviados via mídia eletrônica.

**Resultados:**

O domínio melhor pontuado na avaliação da qualidade de vida para o questionário *Nijmegen* foi o social e para o questionário *World Health Organization Quality of Life* foi o psicológico. As variáveis, gênero, tempo de uso do implante coclear e modalidade auditiva não influenciaram os resultados de ambos os questionários. Apenas a variável nível de instrução correlacionou-se com o domínio meio ambiente do questionário sobre qualidade de vida. A variável compreensão de fala ao telefone associou-se a uma melhor percepção da qualidade de vida para todos os domínios do questionário específico e para a autoavaliação da qualidade de vida em geral.

**Conclusão:**

Na perspectiva dos usuários, o implante coclear trouxe benefícios para os diversos aspectos relacionados à qualidade de vida em ambos os questionários.

## Introduction

Several studies have shown the effectiveness of the cochlear implant (CI) through the assessment of hearing and language skills; however, these tests are limited in terms of the impact of such treatment on social relations, well-being, and the individual's ability for easy communication, all aspects related to the quality of life.

Researchers in the area have been interested in the inclusion of measures that can more fully evaluate the impact of hearing impairment and the available possibilities of habilitation and rehabilitation, using, for this purpose, quality of life measures.

The World Health Organization (WHO) defines quality of life as “the individuals’ perception of their position in life in the context of the culture and value systems in which they live and in relation to their goals, expectations, standards, and concerns.”

The emphasis given in recent years to the subjective and multidimensional aspects related to quality of life was, therefore, derived from the need to understand the impact of a particular complaint and its treatment from the patient's point of view. This allows the analysis of health through different domains, such as physical, functional, social and emotional.^1^

Some tools are available to evaluate the population's quality of life and, among them, the generic questionnaires, used in the general population, without specifying the pathologies, and the specific questionnaires, designed to evaluate the quality of life in a population that has or had a certain disability.^2^

It is necessary to better understand the different aspects related to quality of life of the adult population that uses CI, aiming to obtain more detailed information, which will allow professionals to assist in the CI process, in the validation of the results of this technology, as well as in the management of the therapeutic process.

Therefore, the aim of this study was to assess the quality of life in adult CI users and to verify the associations between age, gender, level of education, hearing status and telephone use for the different aspects related to quality of life.

## Methods

This was a cross-sectional and clinical study that assessed the quality of life in 26 adult users of CI, 14 females and 12 males, aged between 18 and 62 years with mean duration of 80 months of CI use.

The study was approved by the Research Ethics Committee of the institution under opinion n. 442/15. All participants received an invitation letter through electronic mail. After agreeing to participate in the research, they signed the Free and Informed Consent form.

Quality of life assessment was performed using the Nijmegen Cochlear Implant questionnaire (NCIQ-P) and the generic World Health Organization Quality of Life (WHOQOL-bref) questionnaire, accessed and answered online. All the documents (letter of research presentation, informed consent form and NCIQ-P and WHOQOL-bref questionnaires) were made available at the Google Docs platform of online questionnaires.

The following inclusion criteria were used to select the research subjects: age between 18 and 60 years of age; having finished high school as the minimum level of schooling; hearing impairment acquired after the oral language development (post-lingual) and time of CI use ≥12 months.

The NCIQ questionnaire is characterized as a specific questionnaire that assesses quality of life in adult CI users, which was developed by Hinderink et al. (2000)^3^ and adapted to Brazilian Portuguese by Santos et al. (2017, in press).^4^ It consists of 60 questions divided into three overall domains, with their respective subdomains: physical (basic perception of sound, advanced perception of sound and speech production), psychological (self-esteem) and social (limitations in social activities and functions).

The WHOQOL-bref is the abbreviated version of the WHOQOL-100 generic quality of life questionnaire developed by the WHO and validated in Brazil.^5^, ^6^ It consists of 26 questions, two of them about overall quality of life, called “quality of life self-assessment”. The other questions were taken from the WHOQOL-100 and represent each of the 24 tool features, covering four domains: physical, psychological, social relations and environment. Each domain is scored independently, with no overall score for the tool.

The overall quality of life questions, called quality of life self-assessment, represented in this study the mean of the scores obtained in Questions 1 and 2.

The nonparametric Wilcoxon test (variables with two categories) and the nonparametric Kruskal–Wallis test (variables with three categories) were used to associate gender, level of schooling, hearing status (unilateral or bilateral CI) and the use of the telephone with the NCIQ-P and WHOQOL-bref domain scores. Similarly, Spearman's correlation test was used to associate the variables age, and time of CI use with the domain scores of both questionnaires, as well as the analysis of the NCIQ-P and the WHOQOL-bref domains in relation to the quality of life self-assessment and in the analysis of the corresponding domains of the questionnaires.

The variable telephone use was defined based on NCIQ Question 60 scores: patients with a score <50 were classified as “no” and the others were classified as “yes”. The non-parametric Wilcoxon test was used to associate telephone use with the domain scores of both questionnaires.

## Results

The quality of life assessment based on the specific tool NCIQ-P demonstrated that the social domain, comprising the subdomains limitations in social activities and interactions was the best scored feature in adults with post-lingual hearing loss and CI users participating in the study, followed by the psychological and physical domains. For the WHOQOL-bref generic tool, the psychological and physical domains were the best evaluated aspects (Table 1).

**Table 1:** Descriptive measures for the NCIQ-P and WHOQOL-bref domains.

Subdomain/domain	Mean	Standard deviation	Minimum	Median	Maximum
*NCIQ-P*					
Physical	67.8	16.2	35.8	65.0	97.5
Psychological	69.9	20.1	12.5	77.5	90.0
Social	72.9	19.4	6.3	78.1	97.5
Overall	70.2	16.1	20.1	73.6	90.8

*WHOQOL-bref*					
Physical	72.5	14.0	42.9	75.0	96.4
Psychological	73.6	9.9	58.3	72.9	95.8
Social Relations	69.9	15.3	33.3	70.8	100.0
Environment	61.1	12.6	34.4	60.9	87.5
QoL self-assessment	77.4	11.2	50.0	75.0	100.0

The comparison between similar domains in each questionnaire showed that the physical and psychological domains of the WHOQOL-bref questionnaire correlated with the corresponding NCIQ-P domains, with *p* = 0.04 for the physical domain and *p* = 0.01 for the psychological domain. Additionally, the quality of life self-assessment correlated with the overall NCIQ-P score (*p* = 0.02).

Regarding the analyzed variables, gender, duration of CI use and hearing modality did not influence the quality of life results of both questionnaires.

Regarding the stimulation condition, although not statistically significant, the use of bilateral CI seemed to have influenced the quality of life results for the NCIQ-P specific questionnaire, since a higher score was observed in users of bilateral CI for all domains of the tool (Table 2).

**Table 2:** Descriptive measures for NCIQ-P domains and subdomains according to the hearing status (unilateral or bilateral HF).

Subdomain/domain	HS	*M*	DP	Min.	Med.	Max.	*p*
Physical NCIQ	Bilateral	74.9	16.7	49.2	80.0	94.2	0.29
	Unilateral	65.6	15.9	35.8	64.2	97.5	
Self-esteem	Bilateral	71.7	19.0	35.0	76.3	90.0	1.00
	Unilateral	69.4	20.9	12.5	77.5	90.0	
Social NCIQ	Bilateral	78.0	15.0	53.8	76.7	97.5	0.71
	Unilateral	71.3	20.6	6.3	78.8	90.0	
Overall NCIQ	Bilateral	74.8	13.5	55.7	74.0	90.8	0.69
	Unilateral	68.8	16.8	20.1	73.6	90.8	

HS, hearing status; *M*, mean; SD, standard deviation; Min., minimum; Med., median; Max., maximum; *p*, *p*-value.

The variable level of education correlated with the environment domain of the WHOQOL-bref questionnaire (*p* = 0.02).

The complex ability of understanding speech on the telephone was associated with the NCIQ-P, WHOQOL-bref domains and with the WHOQOL-bref quality of life self-assessment. Patients who reported good use of the telephone showed, on average, higher scores in the psychological, social, and global domains of the NCIQ-P, respectively, *p* = 0.015; *p* = 0.02 and *p* = 0.001 (Table 3). For the WHOQOL-bref generic questionnaire, a correlation was found only between telephone use and quality of life self-assessment (*p* = 0.042) (Table 3).

**Table 3:** Association of telephone use with the domains of the questionnaires NCIQ-P and WHOQOL-bref.

Domains	TU	*n*	*M*	SD	Min.	Med.	Max.	*p*
Psychological NCIQ	No	9	57.22	25.72	12.50	60.00	87.50	0.01^a^
	Yes	17	76.62	12.75	35.00	77.50	90.00	
Social NCIQ	No	9	58.99	25.09	6.25	65.00	82.50	0.02^a^
	Yes	17	80.20	10.27	53.75	80.31	97.50	
Overall NCIQ	No	9	56.05	17.63	20.14	60.79	74.03	0.00^a^
	Yes	17	77.65	8.71	55.69	78.89	90.83	
Physical WHOQOL	No	9	70.63	12.60	53.57	75.00	92.86	0.43
	Yes	17	73.53	15.00	42.86	78.57	96.43	
Psychological WHOQOL	No	9	71.30	11.87	58.33	66.67	95.83	0.25
	Yes	17	74.75	8.90	58.33	75.00	95.83	
Social Relations WHOQOL	No	9	62.96	16.20	33.33	66.67	91.67	0.10
	Yes	17	73.53	13.89	50.00	75.00	100.00	
Environment WHOQOL	No	9	56.94	9.21	40.63	59.38	71.88	0.24
	Yes	17	63.24	13.87	34.38	68.75	87.50	
QoL self-assessment WHOQOL	No	9	72.22	5.51	62.50	75.00	75.00	0.04^a^
	Yes	17	80.15	12.55	50.00	75.00	100.00	

QoL, quality of life; TU, telephone use; *n*, subjects; *M*, mean; SD, standard deviation; Min., minimum; Med., median; Max., maximum; *p*, *p*-value.

a Statistical difference *p* ≤ 0.05.

## Discussion

Although all the analyzed studies demonstrated that the population of adult CI users with post-lingual hearing loss showed significant quality of life improvement after CI use, a great variability can be observed regarding the scores.^3^, ^7^, ^8^, ^9^ This is because the term quality of life aggregates different life conditions and circumstances, in order to make it difficult to establish a referential in relation to the score to be obtained in a given population.

In the present study, the domains with the highest scores in the NCIQ-P specific questionnaire were, respectively social, psychological, and physical domains, with very similar scores for the psychological and physical domains (Table 1). These results corroborate the data shown in literature, in which the social domain represents the best assessed aspect related to quality of life by the adult population using CI.^3^, ^4^, ^9^, ^10^, ^11^, ^12^ The study that translated and adapted this tool into Brazilian Portuguese found similar scores for all domains, with the social domain also being the best scored aspect among the study participants.^4^

The higher score obtained for the social domain is possibly associated with the questions that comprise this aspect: limitations of the CI user in several environments and the user's social interaction with different individuals and groups, since these aspects are directly related to the communication and insertion in different daily life situations. The hearing status improvement and, therefore, the improvement in communication situations, certainly represents a positive impact on the users’ socialization.

Regarding the WHOQOL-bref questionnaire, a high score was observed among the present study participants in all domains, with a higher score for the psychological and physical domains, respectively (Table 1).

The values found for each domain were higher than those described for quality of life in the overall population by Cruz et al. (2011).^1^ On the other hand, approximate scores were described in a Brazilian study evaluating quality of life in CI users using this same tool.^13^

The highest score obtained in the CI user population compared to that found in the overall population may suggest a better quality of life perception in CI users, especially regarding the aspects that constitute the physical and psychological domains of the WHOQOL-bref questionnaire. Albrecht and Devlieger (1999)^14^ had already described the “disability paradox”, in which individuals with disabilities can report good or excellent quality of life, so as to reflect how some individuals manage to live with their limitations and value certain aspects of life that go unnoticed by biologically healthy individuals.

The variables gender, time of CI use, and age did not influence the quality of life results of both questionnaires. Other studies in the scientific literature had previously described the little significant influence of gender, age, and time of CI use on the quality of life of adult CI users.^13^, ^15^, ^16^, ^17^

Regarding the assessment of the educational level influence on the research subjects’ quality of life, a correlation was found between this variable and the environment domain of the WHOQOL-bref questionnaire (*p* = 0.02). These results resemble those described by Cruz et al. (2011)^1^ and Angelo et al. (2016),^13^ which showed that the variables level of schooling and socioeconomic level can have an impact on the quality of life of the overall population and, possibly, even more on the quality of life of individuals with hearing impairment, considering the difficulty of having access to rehabilitation, schooling, and work.^18^

Due to its specificity for the CI user population, the NCIQ-P seemed to be more significant to evaluate the influence of bilateral CI on the different aspects related to the quality of life of the assessed population, since even without statistical significance, a better score was found in bilateral CI users in all domains of the questionnaire (Table 2). This trend was not observed in the quality of life assessment when using the generic WHOQOL-bref questionnaire.

The higher score obtained by users of bilateral CI is associated with the benefits of binaural hearing, resulting from the device use in both ears, in order to provide more security and better auditory performance in the different daily life situations. The results of the present study are in agreement with those by Olze et al. (2012),^19^ who also found better quality of life in bilateral CI users, with a higher score for all domains of the NCIQ questionnaire after the second CI.

In the present study, the existing association between the ability to understand speech on the telephone and quality of life was assessed through the analysis of the answers obtained from Question 60 of the NCIQ-P specific questionnaire. Correlations were found between the possibility of maintaining a satisfactory telephone conversation for both the psychological (*p* = 0.015) and social (*p* = 0.020) domains; as well as for the overall quality of life assessment (*p* = 0.001) of the NCIQ-P questionnaire (Table 3).

The ability to understand speech on the telephone did not correlate with the domains of the WHOQOL-bref generic questionnaire. However, in spite of a poor correlation (*p* = 0.042), subjects who were able to maintain a simple telephone conversation showed a more positive assessment of their overall quality of life in the WHOQOL-bref questionnaire.

The strongest associations between the ability to understand speech on the telephone and the different aspects related to quality of life were found for the domains of the NCIQ-P specific questionnaire, thus reinforcing the importance of the use of a specific tool to assess the CI user population, created with the objective of reflecting the achievements and difficulties experienced by these patients in their activities of daily living.

These results suggest that, considering its specificity, the NCIQ-P seemed to be more sensitive in evaluating the influence of the more complex hearing abilities in the different aspects related to quality of life. The data found in the present study corroborate the one carried out by Rumeau et al. (2015),^12^ in which the authors observed that the ability to understand speech on the telephone can impact the overall quality of life estimate in CI users when using the NCIQ-P specific questionnaire.

## Conclusion

From the users’ perspective, CI brought benefits to the different aspects related to quality of life in both questionnaires. The NCIQ-P questionnaire was more favorable to assess quality of life questions related to the communication and interaction of CI users. The combined use of quality of life measures represented a clinical differential capable of complementing the objective evaluation data and guiding the management of the therapeutic process.

## Conflicts of interest

The authors declare no conflicts of interest.
